# Factors Reducing Postoperative Pain Related to Root Canal Treatment: A Narrative Review of Systematic Reviews

**DOI:** 10.3390/dj13030102

**Published:** 2025-02-26

**Authors:** Abdelrahman M. Alhilou

**Affiliations:** Department of Restorative Dentistry, College of Dentistry, Umm Al-Qura University, Makkah 24382, Saudi Arabia; amhilou@uqu.edu.sa; Tel.: +966-53-0648022

**Keywords:** root canal therapies, endodontics, pain, postoperative

## Abstract

**Background/Objectives:** Pain after root canal treatment is a common concern that can greatly affect a patient’s quality of life. Identifying the factors contributing to this pain and focusing on those supported by high-quality research can lead to more effective pain management. This narrative review aims to analyze all available systematic reviews on this topic to determine what has been proven to help decrease pain following the root canal procedure. **Methods:** A comprehensive literature search was conducted across Scopus and Google Scholar from January 2000 to January 2024, using defined MeSH terms. This yielded 51 systematic reviews, of which 45 specifically investigated factors reducing postoperative pain related to root canal treatment. **Results:** Eleven factors were identified in the literature, with only eight factors supported by low- to moderate-quality evidence to reduce postoperative pain related to root canal treatment. These eight factors include (1) laser therapy, (2) nonsteroidal anti-inflammatory drugs (especially when combined with acetaminophen) and corticosteroids, (3) ultrasonic irrigation and low concentrations of sodium hypochlorite, (4) cryotherapy, (5) specific combinations of intracanal medicaments (notably calcium hydroxide with chlorhexidine), (6) bioceramic sealers, (7) rotary instrumentation, and (8) apical patency. **Conclusions:** The insights gained from this narrative review highlight several important factors that reduce postoperative pain related to root canal treatment. Nevertheless, the observed variability in the quality of the evidence calls attention to the necessity for further high-quality research.

## 1. Introduction

Most dental pain arises from pulpal and periapical disease and requires root canal treatment, which is a procedure that aims to address the infection of a tooth by removing the pulp and cleaning and filling the root canal system [1,2]. Postoperative pain related to root canal treatment is a common health issue that can affect a patient’s quality of life [3]. Reports suggest that this pain occurs in 3% to 58% of cases [4,5].

Postoperative pain related to root canal treatment is primarily linked to the inflammatory process often triggered by bacterial toxins from the root canal system into the periapical tissues, leading to acute inflammation. Chemical and mechanical injuries to the periapical tissues also contribute to this inflammatory response [6]. The resulting injury stimulates an increase in the expression and release of neuropeptides from the nerve fibers in the periapical lesion, which may intensify postoperative pain and prolong recovery [7]. It has been noted that during periapical inflammatory reactions, the density of nerve fibers correlates with the size of periapical lesions and the number of inflammatory cells [7,8].

Several factors have been proposed to correlate with postoperative pain related to root canal treatment. These include preoperative pain, missed canals, and apical patency practices, which can influence postoperative discomfort [9,10]. The type and anatomy of the treated tooth, particularly molars in the mandibular arch, are also significant, alongside gender differences, with women reporting more pain, potentially due to hormonal influences [9,11]. Age appears to play a role, with older patients often experiencing less pain [12]. The choice of intracanal medications and the number of treatment visits (single versus multiple) can affect pain levels as well. Furthermore, occlusal adjustments and advanced endodontic techniques may influence pain outcomes, underscoring the multifactorial nature of postoperative pain related to root canal treatment [13].

There is varied evidence on factors that reduce postoperative pain, with some showing promise but lacking strong, conclusive proof. Therefore, this review aims to provide an overview of the current systematic reviews on factors reducing postoperative pain related to root canal treatment, specifically focusing on the reported risks of bias and the quality of evidence. The goal is to help researchers identify areas that require further investigation in future research.

## 2. Materials and Methods

**Figure 1:**
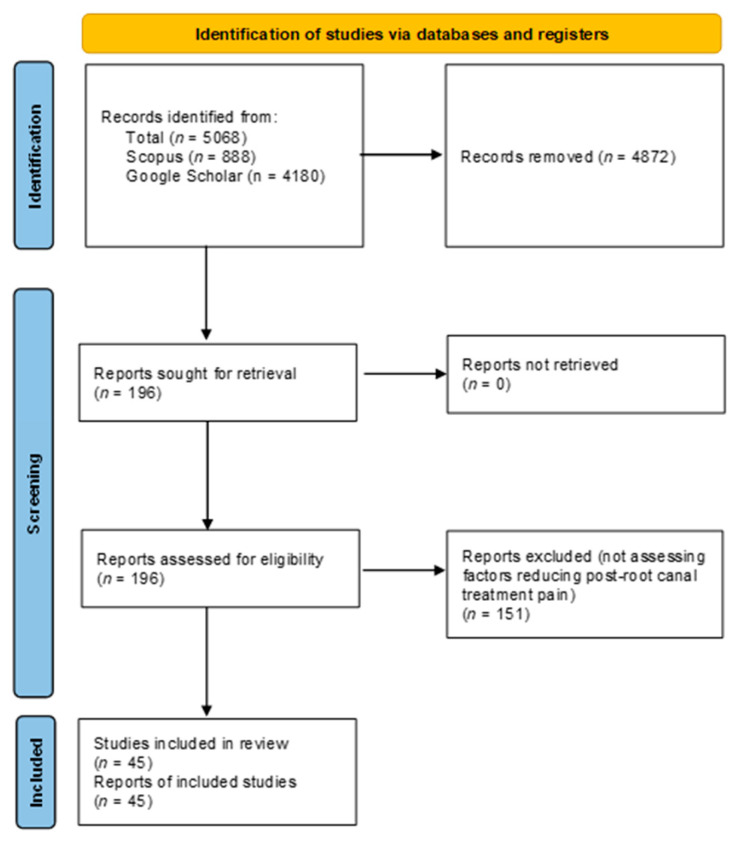
Flowchart illustrating the process of article selection.

A literature search was performed from January 2000 to January 2024 using Scopus and Google Scholar, focusing on the following MeSH terms: (“root canal treatment” OR “endodontic treatment” OR “root canal therapy”) AND (“post-operative pain” OR “post-treatment pain” OR “pain management” OR “flare up”) AND (“systematic review” OR “meta-analysis”). This search identified a total of 5068 papers (*n* = 888 from Scopus and *n* = 4180 from Google Scholar). Only systematic reviews and meta-analyses published in English and focusing on factors reducing postoperative pain following root canal treatment were included. After removing duplicates and screening titles, 196 reviews remained. Abstracts of these reviews were examined, resulting in the inclusion of 51 systematic reviews. After examination of these studies, only 45 specifically investigated factors reducing post-root canal treatment pain (Figure 1).

## 3. Results

The search results for systematic reviews exploring factors reducing postoperative pain related to root canal treatment identified 11 factors. Some of these factors were investigated by multiple systematic reviews, while others were examined by only one. The factors identified in these systematic reviews, along with the corresponding quality of evidence as described in each review, are summarized in Table 1. Out of the 11 factors found, only 8 were proved by low- to moderate-quality evidence to reduce post-root canal treatment pain. No studies reviewed provided high-quality evidence.

## 4. Discussion

This narrative review identified 8 out of 11 factors with low- to moderate-quality evidence that may help reduce pain following root canal treatment. Nevertheless, it is important to note that other factors may exist in the literature, potentially investigated through clinical trials rather than systematic reviews, which should be investigated in future research efforts. Moreover, it is crucial to consider other emerging technologies and methods that show potential benefits. For instance, while the effectiveness of cold atmospheric plasma (CAP) in pain reduction has not been systematically reviewed in the context of postoperative pain, its role in disinfection is promising [60]. As demonstrated by Sanesi et al. (2024), CAP has an effect on bacterial decontamination, especially Enterococcus faecalis. This enhancement in microbial control might indirectly contribute to reducing postoperative complications, including pain, by reducing inflammation [60].

The following section will discuss the impact of each of these 11 factors and the quality of the supporting evidence.

### 4.1. Lasers

Systematic reviews demonstrate a potential analgesic effect of low-level laser therapy (LLLT) following root canal treatment [14,15,16,17,19], retreatment [14,15,16,18], and apical surgery [15]. These studies investigated randomized controlled trials that used various laser techniques, typically targeting the mucosa overlying the apex of the treated tooth, the surgical wound in cases of periapical surgery, or utilizing laser-assisted irrigation techniques. The quality of evidence concerning LLLT for postoperative pain related to root canal treatment varies significantly across studies. Some systematic reviews reported studies with a low risk of bias, while others showed a moderate to high risk with several drawbacks, such as an inconsistency in methodology. For example, differences in laser parameters, the number of treatment sessions, and the timing of laser therapy administration can influence postoperative pain outcomes.

### 4.2. Single and Multiple Visits

One of the systematic reviews found that postoperative pain for single-visit and multiple-visit root canal treatment is similar [20]. Most clinical trials in this review had small sample sizes, suggesting that the current body of evidence may not provide a definitive answer to whether one approach is superior to the other in reducing pain [20]. In contrast, another systematic review reported a higher incidence of postoperative pain associated with single-visit treatments compared to multiple-visit treatments [21]. Despite this finding, the presence of high statistical heterogeneity among randomized controlled trials included in this review diminishes the reliability of the result [21]. Interestingly, two other systematic reviews concluded that there was no significant difference in pain incidence and intensity after root canal treatment between the single and multiple visits [22,23].

### 4.3. Root Canal Sealers

The systematic review by Jamali et al. (2021) [24] highlighted no statistically significant difference in pain scores between the two types of sealers (resin-based and bioceramic) within 24 to 48 h postoperatively. This is consistent with the findings from the reviews conducted by Carr and MacInnes (2022) [26], showing that there were no differences in postoperative pain levels between groups using resin and non-resin sealers. On the other hand, Mekhdieva et al. (2021) [25] found that patients who received bioceramic sealers had significantly less postoperative pain compared to those using traditional sealing methods, particularly at both the 24 and 48 h postoperative time points. It has been suggested that while resin-based sealers do not exacerbate pain, bioceramics could offer improved pain outcomes. Zamparini et al. (2024) [28] also observed a non-significant trend of lower postoperative pain when using bioceramic sealers compared to traditional epoxy resin sealers. Their findings suggest that although the differences are not significant, bioceramic sealers may be slightly better at reducing postoperative pain.

The differences among the studies, such as variations in methodologies, patient selection, types of interventions, and the potential for bias, make it difficult to draw clear conclusions. Importantly, several reviews cited a high risk of bias and inconsistency in the reporting of outcomes [24,26], which may affect the reliability of some findings.

### 4.4. Apical Patency

The study by Xiqian et al. (2024) [29] showed notable differences in mean pain scores on days one and two between the apical patency and non-patency groups, supporting the idea that patency facilitates better drainage and minimizes discomfort. However, the lack of significant differences in the mean number of required analgesic doses suggests that, while patency may reduce pain sensations, some level of analgesic intervention remains necessary for managing postoperative discomfort. The evidence quality was rated low for pain alleviation, indicating the necessity for further high-quality randomized controlled trials to validate these findings and address inconsistencies across studies.

### 4.5. Instruments Used for Root Canal Preparation

The systematic review by Sun et al. (2018) [30] showed that rotary instruments significantly reduce both the incidence and intensity of postoperative pain compared to manual instruments. Furthermore, the comparison between multiple rotary-file systems and reciprocating systems revealed that rotary instruments are superior in reducing pain of teeth diagnosed as having pulpitis or pulp necrosis (with or without periapical lesions) following single-visit root canal treatments compared to manual and reciprocating files. Similarly, Nobar et al. (2021) [31] focused on single-visit root canal therapies, regardless of pulpal and apical diagnosis, and reported higher incidence rates of postoperative pain associated with reciprocating instrumentation compared to rotary instrumentation. However, the differences were not statistically significant across all time intervals examined. This indicates that while there may be trends suggesting higher discomfort with reciprocating systems, they do not achieve statistical significance, and analgesic intake was also similar between the two groups. Moreover, in multi-visit procedures, the findings from Antony et al. (2022) [32] showed no significant differences in postoperative pain levels between continuous and reciprocating instruments, indicating that both types of engine-driven systems are effective at minimizing pain in multi-visit procedures.

### 4.6. EALs and Radiographs

The review by Kaur et al. (2024) [33] found no statistically significant difference in postoperative pain between the EAL group and the radiograph group. This finding suggests that the use of EALs does not result in higher discomfort levels for patients compared to conventional radiographic techniques. However, the certainty of the evidence was very low. Therefore, well-planned randomized controlled trials with standardized methods are necessary.

### 4.7. Medications

Twelve systematic reviews investigated medications used to reduce postoperative root canal treatment pain. Below, the discussion categorizes these medications based on their effectiveness in reducing postoperative pain.

#### 4.7.1. Nonsteroidal Anti-Inflammatory Drugs (NSAIDs)

NSAIDs are the primary choice for managing postoperative pain in endodontic procedures. According to the review by Aminoshariae et al. (2016) [34], these drugs demonstrated moderate evidence for efficacy, particularly ibuprofen at doses of 400 to 600 mg. Shamszadeh et al. (2017) [36] found that NSAIDs were effective in lowering pain scores when compared to a placebo. In another study by Zanjir et al. (2020) [39], it was reported that NSAIDs, whether taken on their own or alongside acetaminophen, helped reduce pain intensity, especially in the first 24 h after starting treatment.

#### 4.7.2. Acetaminophen

Studies indicate that combining NSAIDs with acetaminophen improves pain management outcomes. For example, Aminoshariae et al. (2016) [34] suggested that when NSAIDs alone are ineffective, a combination with acetaminophen can be beneficial. Shahravan and Nekouei (2022) [44] reported that NSAIDs + acetaminophen significantly reduced pain at 6–8 h after treatment.

#### 4.7.3. Corticosteroids

Corticosteroids also showed significant efficacy in managing postoperative endodontic pain. Iranmanesh et al. (2017) [35] showed that glucocorticosteroids were effective in reducing pain, although variability in study outcomes prevented a meta-analysis. Shamszadeh et al. (2018) [38] found that corticosteroids significantly reduced pain scores at various postoperative intervals. Alajlan et al. (2024) [45] concluded that systemic corticosteroids were significantly effective in lowering pain reports at 6, 12, and 24 h, with a reduction in pain intensity as a primary benefit.

There is considerable heterogeneity regarding the routes of corticosteroid administration, the corresponding dosages, and the timing of administration used in managing postoperative pain following endodontic treatment. Upon examining the systematic reviews included in this study, no specific dosage was proposed; however, various randomized controlled trials cited within these reviews indicate a wide range of dosages being used. For oral administration, doses include 20 mg and 30 mg of prednisolone, as well as 4 mg of oral dexamethasone [26]. Local administration methods include intracanal injections of 2.5% prednisolone and 0.1 mL of 4 mg/mL dexamethasone [26]. Reports of dosages of intraligamentary injections range from 4 to 8 mg of methylprednisolone and dexamethasone at 0.2 mL [26]. Furthermore, supraperiosteal injections were reported with the utilization of 4 mg of dexamethasone, while reports of intramuscular administrations vary, with concentrations of 2, 4, 6, or 8 mg/mL [26]. The timing of administration also differs, with some studies suggesting preoperative dosing and others indicating postoperative dosing. The variability in administration routes, dosages, and timings indicates an urgent need for further high-quality research with standardized protocols.

#### 4.7.4. Antibiotics

The role of antibiotics in managing postoperative pain after endodontic treatment has been questioned in several systematic reviews. Milani et al. (2022) [43] found that antibiotic administration following endodontic treatment of symptomatic non-vital teeth had no significant effect on pain severity at 24 h post-treatment. Similarly, Shamszadeh et al. (2021) [41] concluded that prophylactic antibiotics did not effectively reduce pain or flare-up rates. It is mentioned that while antibiotics can be important for preventing infections, their routine use just to manage pain is not supported by strong evidence.

### 4.8. Irrigation Type, Technique, and Concentration

Five systematic reviews investigated the impact of irrigation on pain following root canal treatment. In the study by Martins et al. (2020) [46], the comparison between sodium hypochlorite and chlorhexidine showed no significant differences in postoperative pain levels, suggesting that both solutions are equally effective; however, the limited number and heterogenicity of studies prevents the authors from drawing definitive conclusions. Adam and Wootton (2022) [47] found that ultrasonic irrigation significantly reduced pain at various points after root canal treatment. This aligns with the findings of Chalub et al. (2022) [48], who reported less postoperative pain associated with ultrasonic irrigation, particularly in cases involving non-vital pulps. This can be explained by several key mechanisms. Firstly, ultrasonic activation generates acoustic microstreaming and cavitation, which disrupts biofilms and displaces debris and bacteria in the root canal system, thereby improving the cleaning process of the root canal. Consequently, it reduces inflammation, which is a critical factor contributing to patient discomfort and pain [61]. Additionally, ultrasonic irrigation can promote better penetration of the irrigant into complex canal anatomies, ensuring that toxins and debris are effectively removed from areas that are challenging to reach with conventional irrigation methods [61].

Regarding irrigant concentration, Prasad et al. (2024) [50] revealed that lower concentrations (≤3%) of sodium hypochlorite were significantly associated with fewer reports of postoperative pain compared to higher concentrations (≥5%) at both 24 and 48 h. This difference may be due to lower concentrations exhibiting less cytotoxicity, leading to reduced tissue damage and inflammation, which can help decrease pain. While higher concentrations of sodium hypochlorite are effective in dissolving pulp tissues and providing antimicrobial benefits, they also carry an increased risk of nerve and tissue irritation, which can result in greater postoperative discomfort [62].

### 4.9. Occlusal Reduction

Two systematic reviews that studied the impact of occlusal reduction on postoperative pain were found in the literature. Nguyen-Nhon et al. (2020) [51] found that occlusal reduction reduces postoperative pain in teeth diagnosed with irreversible pulpitis and symptomatic apical periodontitis, particularly noted at the six-day follow-up. The authors note that reducing occlusion has little impact on pain relief within the first 24 h post-treatment. However, more high-quality research was recommended. Similarly, Shamszadeh et al. (2020) [52] found that there is not enough evidence to support the idea that reducing occlusion helps with pain relief in the first 48 h after treatment. However, they noted that patients often experience significant pain relief by the third day after the procedure. The authors mentioned that this conclusion must be approached with caution because of differences in methods and the limited number of studies analyzed.

### 4.10. Cryotherapy

Three systematic reviews focused on the role of intracanal cryotherapy in reducing postoperative pain following non-surgical endodontic treatment, specifically primary procedures rather than retreatments. Sadaf et al. (2020) [53] highlight that intracanal cryotherapy, specifically using cold saline irrigation at temperatures of 2.5 °C as a final irrigant, significantly reduced pain intensity at 6 and 24 h post-treatment. However, there was no significant reduction in pain at the 48 and 72 h postoperative time points. This suggests that the benefits of cryotherapy may be more immediate rather than lasting over time. Almohaimede and Al-Madi (2021) [54] showed a significant reduction in postoperative pain across multiple time points, specifically at 6, 24, 48, and 72 h following root canal treatment. While the study identified the efficacy of cold irrigation, the reported evidence was categorized as low quality, emphasizing the need for further well-designed randomized clinical trials. Monteiro et al. (2021) [55] also assessed postoperative pain related to primary non-surgical endodontic treatment and concluded that intracanal cryotherapy resulted in lower pain scores at 6 and 24 h following the procedure. However, they noted no significant differences in pain intensity at 48 and 72 h, aligning with the findings of Sadaf et al. (2020) [53]. Therefore, while cryotherapy may effectively reduce direct postoperative pain, its longer-term advantages remain uncertain.

### 4.11. Intracanal Medicament

Four systematic reviews were found in the literature focusing on the role of intracanal medicaments in managing postoperative pain following primary endodontic treatment. Ahmad et al. (2021) [56] assessed the role of calcium hydroxide compared to no dressing or other intracanal medicaments, indicating that calcium hydroxide significantly improved postoperative pain at 24 h, particularly in patients with apical periodontitis. However, they noted that combination therapies, particularly those using steroids or antibiotics, have been found to be more effective than calcium hydroxide alone. This highlights the importance of combining intracanal medicaments to reduce postoperative endodontic pain. Wagh et al. (2022) [57] also found that the use of intracanal medicaments does not lead to increased pain occurrence and may help reduce inter-appointment pain. Alkhamsan et al. (2023) [58] investigated the effectiveness of using antibiotics and steroids as intracanal medicaments. Their findings indicated that this combination can notably enhance pain relief, particularly for patients suffering from symptomatic irreversible pulpitis. Lastly, Hegde et al. (2023) [59] compared calcium hydroxide with other intracanal medicaments, finding that while calcium hydroxide is effective on its own, its effectiveness increases when used in combination with medicaments such as chlorhexidine.

## 5. Conclusions

The current evidence identified 11 factors affecting postoperative pain related to root canal treatment. Interestingly, eight of these factors seem to have a positive effect on pain outcomes, though the quality of evidence supporting each one varies. Laser techniques, NSAIDs combined with acetaminophen and corticosteroids, ultrasonic irrigation, lower sodium hypochlorite concentrations, intracanal cryotherapy, and combining calcium hydroxide with chlorhexidine (as intracanal medicament) all demonstrate low- to moderate-quality evidence for pain reduction. Maintaining apical patency suggests potential pain reduction but is supported by low-quality evidence. Rotary motion versus reciprocation during instrumentation and bioceramic versus resin-based sealers also have potential pain reduction effects, but with inconsistent evidence. Overall, no studies reviewed provided high-quality evidence, emphasizing the need for further high-quality research.

## Figures and Tables

**Table 1:** This table includes titles of systematic reviews that investigated various factors influencing postoperative pain related to root canal treatment. It summarizes the types of included studies, the total number of studies in each review, the reported risk of bias for each study, and the overall quality of evidence assessed by the systematic reviews.

Factors	Titles of Systematic Reviews	Types of Included Studies	Number of Included Studies	Risk of Bias (RB)	Quality of Evidence
(1) Laser	(1) Efficacy of low-level laser therapy in pain management after root canal treatment or retreatment: a systematic review [14]. (2) Effect of low-level laser therapy on postoperative endodontic pain: An updated systematic review [15]. (3) Post-endodontic pain evaluation after different intracanal laser assisted disinfection techniques. A Systematic Review [16]. (4) The influence of laser-activated irrigation on post-operative pain following root canal treatment: A systematic review [17]. (5) A systematic review and meta-analysis on the effects of phototherapy on postoperative pain in conventional endodontic reintervention [18]. (6) Effect of photobiomodulation on postoperative endodontic pain: A systematic review of clinical trials [19].	(1) Randomized controlled trials. (2) Randomized controlled trials. (3) Randomized controlled trials. (4) Randomized controlled trials. (5) Clinical studies. (6) Randomized controlled trials and prospective studies.	(1) 7 (5 related to root canal treatment (RCT) and 2 related to RE-RCT). (2) 12 (6 related to RCT, 2 related to RE-RCT, and 4 periapical surgery). (3) 12 (10 related to RCT, 2 related to RE-RCT). (4) 6, all related to RCT. (5) 5, all related to RE-RCT. (6) 9, all related to RCT.	(1) 6 with moderate RB and 1 with low RB. (2) 7 with low RB, 4 studies with unclear RB, and 1 with high RB. (3) 7 with moderate RB and 5 with low RB. (4) 5 with high RB and 1 with some concerns. (5) All with low risk of bias. (6) Did not conduct an assessment of RB.	(1) Moderate. (2) Low to moderate. (3) Low to moderate. (4) Low to moderate. (5) Low to moderate. (6) Low to moderate.
(2) Single and multiple visits	(1) A systematic review of nonsurgical single-visit versus multiple-visit endodontic treatment [20]. (2) The Comparison of Short-Term Postoperative Pain in Single- versus Multiple-Visit Root Canal Treatment: A Systematic Review and Meta-Analysis Study [21]. (3) The incidence and intensity of postendodontic pain and flareup in single and multiple visit root canal treatments: A systematic review and meta-analysis [22]. (4) Revelation of Outcome of Single visit V/s Multiple visit Endodontic Therapy [23].	(1) Clinical trials. (2) Randomized controlled trials. (3) Randomized controlled trials. (4) Randomized controlled trials.	(1) 29. (3) 27. (4) 21. (5) 22.	(1) Did not conduct an assessment of RB. (2) No significant publication bias. (3) Moderate RB. (4) Moderate RB.	(1) Low to moderate. (2) Moderate. (3) Moderate. (4) Moderate.
(3) Root canal sealers	(1) Evaluation of the effect of the intensity and occurrence of postoperative pain of resin-based and bioceramic root canal sealers: A systematic review and meta-analysis of randomized controlled trial studies [24]. (2) Postoperative pain following root canal filling with bioceramic vs. Traditional filling techniques: A systematic review and meta-analysis of randomized controlled trials [25]. (3) Do resin-based sealers increase post-operative pain following root-canal treatment? [26]. (4) A Systematic Review on Comparison of Periapical Healing and Post-Operative Pain between Bioceramic and Epoxy Resin Based Sealers [27]. (5) The efficacy of premixed bioceramic sealers versus standard sealers on root canal treatment outcome, extrusion rate and post-obturation pain: A systematic review and meta-analysis [28].	(1) Randomize controlled trials. (2) Randomized controlled trials. (3) Not specified. (4) Clinical studies and randomized controlled trials. (5) Prospective clinical trials and randomized clinical trials.	(1) 4. (2) 9. (3) 6. (4) 7. (5) 6.	(1) Low RB. (2) 1 study had a low RB, 6 had moderate RB, and 2 had high RB. (3) High and low RB. (4) Not specified; only the level of evidence is mentioned. (5) Generally, studies had some quality concerns.	(1) Low to Moderate. (2) Moderate. (3) Moderate. (4) Moderate. (5) Moderate.
(4) Apical patency	(1) The effect of apical patency on postoperative pain following endodontic therapy: A systematic review and meta-analysis [29].	(1) Randomized controlled trials and prospective clinical studies.	(1) 4.	(1) 3 studies had high RB and 2 had low RB.	(1) “Low” for pain alleviation and “moderate” for reduced incidence.
(5) Instruments for root canal preparation	(1) Pain after root canal treatment with different instruments: A systematic review and meta-analysis [30]. (2) Effect of rotary and reciprocating instrumentation motions on postoperative pain incidence in non-surgical endodontic treatments: A systematic review and meta-analysis [31]. (3) Post-Endodontic Pain with Different Engine-Driven Endodontic Instruments in Multi-Visit Root Canal Therapy A Systematic Review and Meta-Analysis [32].	(1) Randomized or prospective or retrospective controlled trials. (2) Randomized controlled trials. (3) Randomized controlled trials.	(1) 9 studies were included in the first part when comparing hand files to rotary files. 12 studies were included in the second part of the study when comparing rotation to reciprocation motion. (2) 19. (3) 2.	(1) 16 studies showed a moderate RB and 1 showed a high RB. (2) Studies with a high overall risk of bias were excluded from the meta-analysis. 12 studies had a low RB, 4 had an unclear risk, and 3 had a high RB. (3) Low RB.	(1) Moderate. (2) Moderate. (3) Low.
(6) Electronic apex locators (EALs) and radiographs	(1) Efficacy of electronic apex locators in comparison with intraoral radiographs in working length determination- a systematic review and meta-analysis [33].	(1) Randomized controlled trials.	(1) 11.	(1) 5 studies had high susceptibility to bias and the other 6 trials had unclear susceptibility to bias because they each had at least two unclear bias domains.	(1) Low.
(7) Medications	(1) Evidence-based recommendations for analgesic efficacy to treat pain of endodontic origin: A systematic review of randomized controlled trials [34]. (2) Effect of corticosteroids on pain relief following root canal treatment: A systematic review [35]. (3) The efficacy of non-narcotic analgesics on post-operative endodontic pain: A systematic review and meta-analysis: The efficacy of non-steroidal anti-inflammatory drugs [36]. (4) Effects of Ibuprofen Compared to Other Premedication Drugs on the Risk and Intensity of Postendodontic Pain: A Systematic Review [37]. (5) Efficacy of Corticosteroids on Postoperative Endodontic Pain: A Systematic Review and Meta-analysis [38]. (6) Efficacy and Safety of Postoperative Medications in Reducing Pain after Nonsurgical Endodontic Treatment: A Systematic Review and Network Meta-analysis [39]. (7) Medications Used for Prevention and Treatment of Postoperative Endodontic Pain: A Systematic Review [40]. (8) Effects of antibiotic administration on post-operative endodontic symptoms in patients with pulpal necrosis: A systematic review and meta-analysis [41]. (9) Analgesic efficacy of corticosteroids and nonsteroidal anti-inflammatory drugs through oral route in the reduction of postendodontic pain: A systematic review [42]. (10) The effect of antibiotic use on endodontic post-operative pain and flare-up rate: a systematic review with meta-analysis [43]. (11) What is the best effective postoperative medication in reducing pain after non-surgical root canal treatment? [44]. (12) Systemic Corticosteroid Uses in Endodontics—Part 1: Managing Postoperative Pain [45].	(1) Randomized controlled trials. (2) Randomized controlled trials. (3) Randomized controlled trials. (4) Randomized controlled trials. (5) Randomized controlled trials. (6) Randomized controlled trials. (7) Randomized controlled trials. (8) Randomized controlled trials. (9) Randomized controlled trials. (10) Randomized controlled trials. (11) Randomized controlled trials. (12) Clinical trials.	(1) 21. (2) 18. (3) 27. (4) 7. (5) 18. (6) 11. (7) 10. (8) 8. (9) 5. (10) 6. (11) 11. (12) 25.	(1) Moderate RB. (2) Did not conduct an assessment of RB. (3) Satisfactory RB. (4) High to unclear RB. (5) Low to moderate RB. (6) 5 studies were graded as low RB, 5 as moderate RB, and 1 as high RB. (7) Not specified. (8) Unclear RB. (9) 1 low RB, 1 high RB, and the rest were considered to have unclear RB. (10) Unclear RB and low-quality evidence. (11) 5 studies had low RB, 5 had moderate RB, and 1 had high RB. (12) Not specified	(1) Moderate. (2) Low to moderate. (3) Moderate. (4) Low. (5) Moderate. (6) Moderate. (7) Moderate. (8) Moderate. (9) Moderate. (10) Low. (11) Low to moderate. (12) Moderate.
(8) Irrigation	(1) Post-operative pain after using sodium hypochlorite and chlorhexidine as irrigation solutions in endodontics: Systematic review and meta-analysis of randomised clinical trials [46]. (2) Conventional vs ultrasonic irrigation—which leads to less post-operative pain? [47]. (3) Postoperative pain in root canal treatment with ultrasonic versus conventional irrigation: a systematic review and meta-analysis of randomized controlled trials [48]. (4) Does ultrasonic activation of irrigation during endodontic therapy improve the clinical and microbiological effects? [49]. (5) Sodium Hypochlorite Concentration and Postendodontic Pain—Unveiling the Optimal Balance: A Systematic Review and Meta-Analysis [50]	(1) Randomized controlled trials. (2) Randomized controlled trials. (3) Randomized controlled trials. (4) Randomized controlled trials. (5) Randomized controlled trials.	(1) 3, with 2 contributing to the meta-analysis. (2) 6, with 4 contributing to the meta-analysis. (3) 6, with 4 contributing to the meta-analysis. (4) 4, with 3 contributing to the meta-analysis. (5) 5.	(1) Low RB. (2) Low RB. (3) Low RB. (4) High RB. (5) 1 study had high RB, five had low RB, and 4 had some concerns.	(1) Low. (2) Low. (3) Moderate for 24 and 48 h, and low for 6 h and beyond 48 h. (4) Low. (5) Moderate.
(9) Occlusal reduction	(1) Effect of occlusal reduction on postendodontic pain: A systematic review and meta-analysis of randomised clinical trials [51]. (2) Does occlusal reduction reduce post-endodontic pain? A systematic review and meta-analysis [52].	(1) Randomized controlled trials. (2) Randomized controlled trials.	(1) 7, with 3 contributing to the meta-analysis. (2) 6.	(1) 3 studies had low RB, 3 had some concerns, and 1 had high RB. (2) Low RB.	(1) Low. (2) Low.
(10) Cryotherapy	(1) Effectiveness of Intracanal Cryotherapy in Root Canal Therapy: A Systematic Review and Meta-analysis of Randomized Clinical Trials [53]. (2) Is intracanal cryotherapy effective in reducing postoperative endodontic pain? An updated systematic review and meta-analysis of randomized clinical trials [54]. (3) Effect of intracanal cryotherapy application on postoperative endodontic pain: a systematic review and metaanalysis [55].	(1) Randomized controlled trials. (2) Randomized controlled trials. (3) Randomized controlled trials.	(1) 8. (2) 16, with 7 contributing to the meta-analysis. (3) 8, with 6 contributing to the meta-analysis.	(1) Moderate RB. (2) 6 studies had high RB, 4 had some concerns, and 6 had low RB. (3) 1 study had high RB, 3 had unclear RB, and 4 had low RB.	(1) Moderate. (2) Low. (3) Low to high.
(11) Intracanal medicament	(1) Calcium hydroxide as an intracanal medication for postoperative pain during primary root canal therapy: A systematic review and meta-analysis with trial sequential analysis of randomised controlled trials [56]. (2) Prevalence of endodontic flare-up following intracanal medicament placement in permanent teeth undergoing endodontic treatment—A systematic review [57]. (3) Efficacy of Antibiotics and Steroids as Intra-Canal Medicament in Endodontics: A Systematic Review [58]. (4) Comparative evaluation of calcium hydroxide and other intracanal medicaments on postoperative pain in patients undergoing endodontic treatment: A systematic review and meta-analysis [59].	(1) Randomized controlled trials. (2) Randomized controlled trials. (3) Case-control and randomized controlled studies. (4) Randomized controlled trials.	(1) 18. (2) 17. (3) 9. (4) 9.	(1) Moderate RB. (2) 8 studies had high RB, and 9 had low RB. (3) 6 studies had high RB, and 3 had low RB. (4) 4 studies had high RB, 2 had unclear RB, and 3 had low RB.	(1) Moderate. (2) Not specified. (3) Not specified. (4) Not specified.

Abbreviations are defined as follows: RCT (root canal treatment), RE-RCT (Root Canal Retreatment), RB (risk of bias).

## Data Availability

The data analyzed in this narrative review are derived from previously published systematic reviews, which are accessible through publicly available databases such as Scopus and Google Scholar. Detailed references to these systematic reviews can be found in the manuscript.

## Abbreviations

The following abbreviations are used in this manuscript:

RCT	root canal treatment
RB	risk of bias
LLLT	low-level laser therapy
NSAIDs	nonsteroidal anti-inflammatory drugs
EALs	electronic apex locators
